# Exploring the excited state behavior for 2-(phenyl)imidazo[4,5-c]pyridine in methanol solvent

**DOI:** 10.1038/s41598-017-12146-4

**Published:** 2017-09-15

**Authors:** Dapeng Yang, Min Jia, Jingyuan Wu, Xiaoyan Song

**Affiliations:** 1 College of Mathematics and Statistics, North China University of Water Resources and Electric Power, Zhengzhou, 450046 China; 2 State Key Laboratory of Molecular Reaction Dynamics, Theoretical and Computational Chemistry, Dalian Institute of Chemical Physics, Chinese Academy of Sciences, Dalian, 116023 China; 3 Measurement and Testing Institute of Mongolian Autonomous County of Fuxin, Fuxin, 123100 China

## Abstract

In this present work, we theoretically investigate the excited state mechanism for the 2-(phenyl)imidazo[4,5-c]pyridine (PIP-C) molecule combined with methanol (MeOH) solvent molecules. Three MeOH molecules should be connected with PIP-C forming stable PIP-C-MeOH complex in the S_0_ state. Upon the photo-excitation, the hydrogen bonded wires are strengthened in the S_1_ state. Particularly the deprotonation process of PIP-C facilitates the excited state intermolecular proton transfer (ESIPT) process. In our work, we do verify that the ESIPT reaction should occur due to the low potential energy barrier 8.785 kcal/mol in the S_1_ state. While the intersection of potential energy curves of S_0_ and S_1_ states result in the nonradiation transition from S_1_ to S_0_ state, which successfully explain why the emission peak of the proton-transfer PIP-C-MeOH-PT form could not be reported in previous experiment. As a whole, this work not only put forward a new excited state mechanism for PIP-C system, but also compensates for the defects about mechanism in previous experiment.

## Introduction

Just because of its significance in natural world, hydrogen bond has drawn great attention on the relevant topics^1–3^. Particularly, excited state intramolecular and intermolecular hydrogen bond dynamics, elaborating properties involved in hydrogen bond in the excited state, plays important roles in many photo-physical and photochemical processes, such as photo-induced electron transfer (PET), intra- or inter- molecular charge transfer (ICT), fluorescence resonance energy transfer (FRET), and so forth^4–15^. As one of the fast and quite complex reactions involved in hydrogen bond, the excited state intra- or inter- molecular proton transfer (ESIPT) is considered to be one of the most fundamental and important processes in chemistry, biology and materials^16–22^. Since half a century ago, the ESIPT process was firstly reported by Weller and co-workers in experiment with methylsalicylate^23^, it has been a popular research^24–28^. The proton-transfer tautomerization form (normally named keto in the S_0_ state and keto* in the S_1_ state) has charge redistribution characteristic, which are very fascinating towards the application of laser dyes, UV filters, fluorescence chemosensors, molecular switch, and so on^29–38^. Furthermore, some molecules are highly sensitive to the change in the microenvironments, based on which Sytnik *et al*. reported how the ESIPT chromophores can be used in the study of protein conformations and binding site polarity^39^. Indeed, a lot of spectroscopic techniques were applied to study the ESIPT process in recent years, however, only some indirect information about photochemical and photophysical properties could be provided through experimental investigations, and thus the explanation of ESIPT mechanism still have numerous challenges^40^.

As far as we know, generally speaking, molecules possessing proton donor (O-H or N-H) or acceptor (carbonyl oxygen or aromatic nitrogen) might undergo the ESIPT process upon electronic excitation^4–15^. That is to say, a molecule with a proton donor and an acceptor in close proximity may exist ESIPT reaction, yielding a photo-tautomer of the original molecule. While if the groups involved in the proton transfer reaction are far from each other or without the appropriate geometry to form an intramolecular hydrogen bond, the molecules might still undergo ESIPT with the assistance of solvent molecules^41–47^. This kind of reaction happens because solvent molecules could act as bridges between proton donor and acceptor. Particularly, different hydrogen bond patterns between the target molecule and solvent molecules have decisive effects on the ESIPT reaction.

Recently, as a kind of biologically active system, 2-(phenyl)imidazo[4,5-c]pyridine (PIP-C) has been tested to be the inhibitor for Aurora-A, Aurora-B and Aurora-C kinases^48–50^, which have been also certified to be good probes for microenvironment. Krishnamoorthy and co-workers synthesized and investigated PIP-C and its analogues experimentally. They found that single emission phenomenon could be found just in polar protic methanol (MeOH) solvent, which is different from PIP-C analogues (2-(4′-N,N-Dimethylaminophenyl)imidazo[4,5-b]pyridine (DMAPIP-b) and 2-(4′-N,N-dimethylaminophenyl)imidazo[4,5-c]pyridine) (DMAPIP-c)^49,50^. Therefore, it could be found that the excited state dynamical mechanism of PIP-C is different from these two molecules above. Krishnamoorthy and co-workers mainly focused on PIP-C analogues, and they lose sight of PIP-C itself in previous work. In effect, the detailed study about PIP-C itself is very meaningful not only in biological aspects, but also in photochemical and photo-physical fields. Now that PIP-C is similar with DMAPIP-b and DMAPIP-c, whether the proton transfer process also exists in the excited state? If that is the case, why the second fluorescence could not be detected? Furthermore, it is well known that spectroscopic techniques, such as steady state absorption and fluorescence spectra, time-resolved fluorescence spectroscopy, and so forth, can only provide indirect information about some photochemical and photo-physical properties^41–47^. The specific mechanism still depend on quantum chemical calculations.

**Figure 1:**
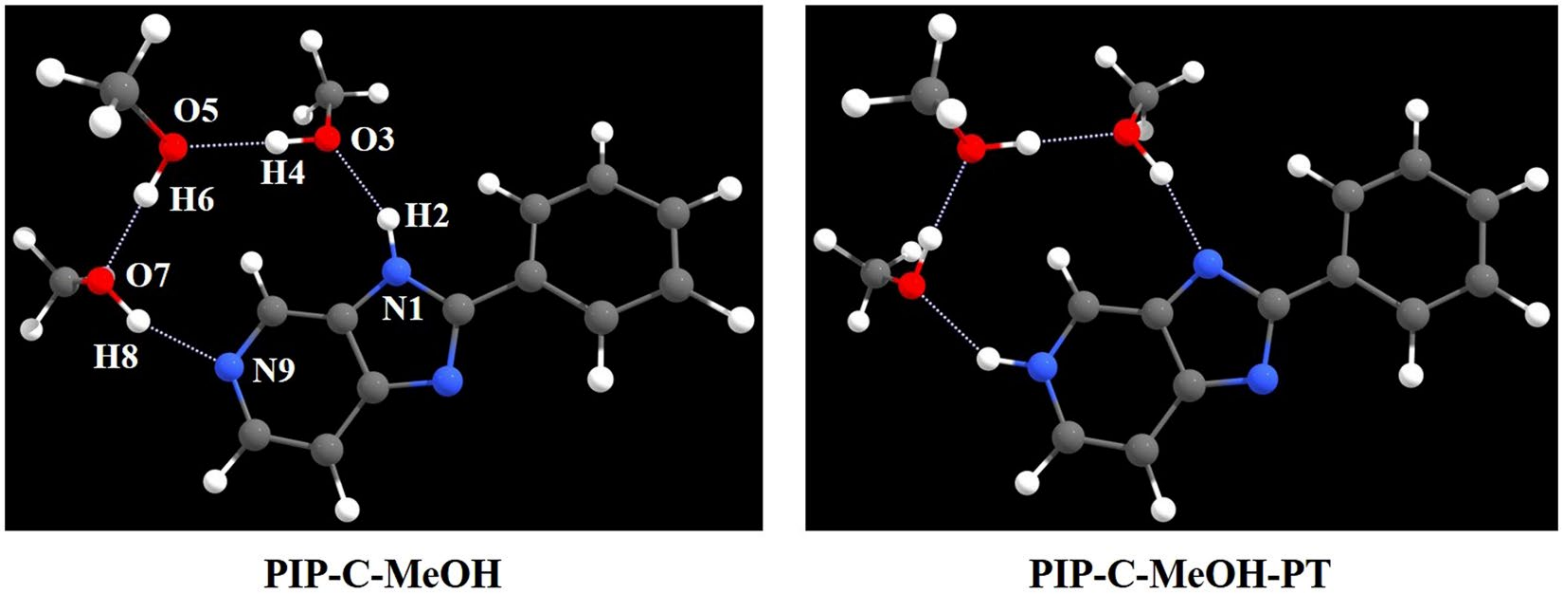
Views of the optimized structures of PIP-C-MeOH and the proton-transfer structure PIP-C-MeOH-PT based on TDDFT/B3LYP/TZVP theoretical level.

In order to further understand the excited state reaction mechanism about PIP-C in MeOH solvent, therefore, a detailed quantum chemical computational investigation has been adopted to study PIP-C systems in this present work. As mentioned in ref^50^, the structures of PIP-C combined with MeOH has been shown in Fig. 1. Given fundamental chemical structure stability, we deem that three MeOH solvent molecules should be the best choice to keep the stable structures. In this present work, we put forward a new excited state mechanism for PIP-C molecule based on density functional theory (DFT) and time-dependent density functional theory (TDDFT) methods.

The remainder of our paper has be organized such that the next section describes the theoretical method. The section 3 shows the discussions about the results of PIP-C calculations including geometries analyses, infrared (IR) vibrational analyses, electronic spectra, charge redistribution and the mechanism analysis. At last, a final section summarizes and provides the conclusion of this present work.

## Theoretical Method

In this present work, all the quantum chemical calculations have been performed via density functional theory (DFT) and time-dependent density functional theory (TDDFT) methods with the Becke’s three-parameter hybrid exchange function with the Lee-Yang-Parr gradient-corrected correlation functional (B3LYP)^51–53^ in combination with the triple-ζ valence quality with one set of polarization functions (TZVP)^54^ basis set in Gaussian 09 program^55^. Before designating functional, we test a number of functionals, including B3LYP (the percentage of Hartree-Fork exchange is 20%), PBEPBE (25%)^56^, MPW1PW91 (42.8%)^57^ and M062X (54%)^58^, among which the B3LYP one provides the most satisfactory agreement with experimental results. In addition, MeOH has been selected as the solvent in our calculations based on the polarizable continuum model (PCM) using the integral equation formalism variant (IEFPCM)^59,60^, which considers the solute in a cavity of overlapping solvent (with an average area of 0.4 Å) with apparent charges to reproduce the electrostatic potential due to the polarized dielectric within the cavity. All the initial geometries were generated from the standard geometrical parameters with minimization without any constraints for the symmetry, bond lengths, bond angles and dihedral angles to obtain the true minima based on DFT and TDDFT. IR vibrational frequencies were calculated at the same level to confirm the absence of imaginary modes. The calculations of vertical excitation energies were also performed from the ground-optimized structures based on TDDFT with IEFPCM (MeOH), and our theoretical calculations predicted the six low-lying absorbing transitions. To illustrate the excited state mechanism for PIP-C system, all the stationary points along the reaction coordinate have been scanned by constraining optimizations to obtain the thermodynamic corrections in the corresponding electronic states. Zero-point energy corrections and thermal corrections to Gibbs free energy were also carried out according to the harmonic vibrational frequencies.

## Results and Discussion

As mentioned above, the geometry optimizations of PIP-C combined with MeOH molecules have been optimized based on DFT and TDDFT methods. Two stable configurations were found (i.e. the structures of PIP-C-MeOH and the proton-transfer structure PIP-C-MeOH-PT) as shown in Fig. 1. To expediently explain the complicated mechanism, the key atoms were marked by a serial number (see Fig. 1). Within the framework of AIM theory (manly based on the analysis of electron density at the specific point (ρ (r))), identification of a critical point (CP) and the existence of a bond path in equilibrium geometry are necessary and sufficient conditions for assigning an interaction between two primary atoms^61,62^. And the AIM analysis of the title compounds ensure the presence of an appreciable interaction between the atoms concerned. The relevant AIM topological parameters involved in the optimized geometries demonstrate that the ρ (r) at the bond critical point (BCP) for PIP-C-MeOH system are close to 0.04 a.u. (the maximum threshold value proposed by Popelier to ensure the presence of hydrogen bond^61,62^. What is more, the corresponding ∇^2^ ρ_c_ values are also in the range (0.02~0.15 a.u.)^61,62^. Therefore, we can confirm that all these intermolecular hydrogen bonds should be formed for PIP-C-MeOH complex. Herein, we list the most important bond lengths and bond angles of PIP-C-MeOH and PIP-C-MeOH-PT in Table 1. From this table, it could be clearly found that the N1-H2 changes from S_0_-state 1.003 Å to S_1_-state 1.025 Å, meanwhile, hydrogen bond H2···O3 shortens from 1.924 Å to 1.878 Å upon the photo-excitation. In addition, given the bond angle, δ(N1-H2-O3) also increases from 150.4° to 154.2°. Compared with other hydrogen bonds, N1-H2···O3 owns the biggest change. According to previous work^4–13^, we can say N1-H2···O3 should be strengthened in the S_1_ state. Just due to this kind of strengthening tendency, the ESIPT might firstly happen along N1-H2···O3 in the S_1_ state. By contrary, it should be noticed that the hydrogen bond O7-H8···N9 is weakened based on excitation, which reveals that the ESIPT process might not occur firstly along this hydrogen bond wire.

**Table 1:** The primary bond lengths (Å) and bond angles (°) of PIP-C-MeOH and PIP-C-MeOH-PT forms in both S_0_ and S_1_ states.

Electronic state	PIP-C-MeOH		PIP-C-MeOH-PT	
	S_0_	S_1_	S_0_	S_1_
N1-H2	1.003	1.025	1.857	1.896
H2-O3	1.924	1.878	0.988	0.985
O3-H4	0.981	0.982	1.796	1.805
H4-O5	1.818	1.809	0.983	0.982
O5-H6	0.983	0.983	1.828	1.820
H6-O7	1.803	1.806	0.981	0.981
O7-H8	0.985	0.984	1.952	1.942
H8-N9	1.876	1.895	1.024	1.025
δ(N1-H2-O3)	150.4°	154.2°	167.4°	166.8°
δ(O3-H4-O5)	176.8°	176.6°	178.6°	179.0°
δ(O5-H6-O7)	177.4°	178.2°	175.3°	176.6°
δ(O7-H8-N9)	159.1°	158.7°	141.9°	142.8°

**Figure 2:**
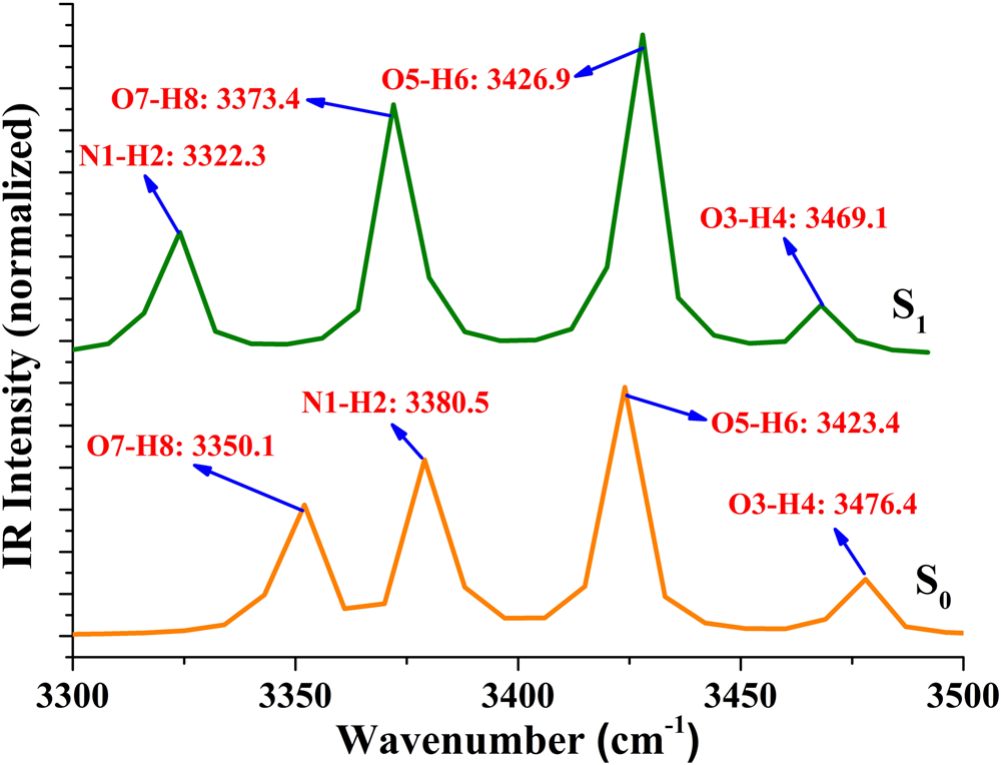
The theoretical IR spectra of PIP-C-MeOH structure in MeOH solvent at the spectral region of corresponding chemical bonds in both S_0_ and S_1_ states.

Furthermore, it is well-known that the red-shift or blue-shift of chemical bonds could be also a manner to predict the changes of excited state hydrogen bond, as proposed by Han *et al*.^4–8^. In view of this, we also calculated the infrared (IR) vibrational spectral shift in this work. We show the vibrational spectra of PIP-C-MeOH system in conjunct vibrational region of N1-H2, O3-H4, O5-H6 and O7-H8 bonds in Fig. 2. One should be mentioned that our theoretical N1-H2 stretching vibrational frequency in the S_0_ state is strongly downshifted by 58 cm^−1^ from 3380.5 cm^−1^ to 3322.3 cm^−1^, which confirms the strengthening of hydrogen bond N1-H2···O3 in the S_1_ state. In consideration of O7-H8···N9, a blue-shift has been found from S_0_-state 3350.1 cm^−1^ to S_1_-state 3373.1 cm^−1^. Obviously, O7-H8···N9 is weaken upon the photo-excitation, which is consistent with the results of structural analyses above. Once again we verify that photo-excitation process could result in the strengthening of hydrogen bond N1-H2···O3 as mentioned above, based on which the ESIPT process is possible to occur in the excited state.

**Figure 3:**
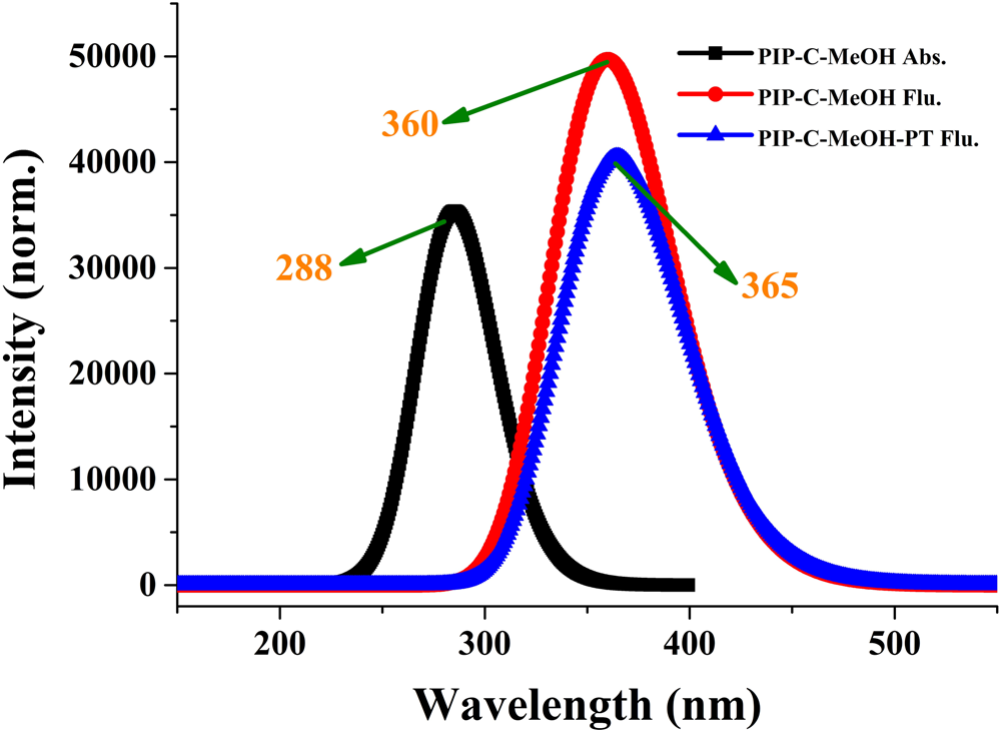
Our calculated absorption and emission spectra of PIP-C-MeOH and PIP-C-MeOH-PT complexes based on TDDFT/B3LYP/TZVP theoretical level.

**Figure 4:**
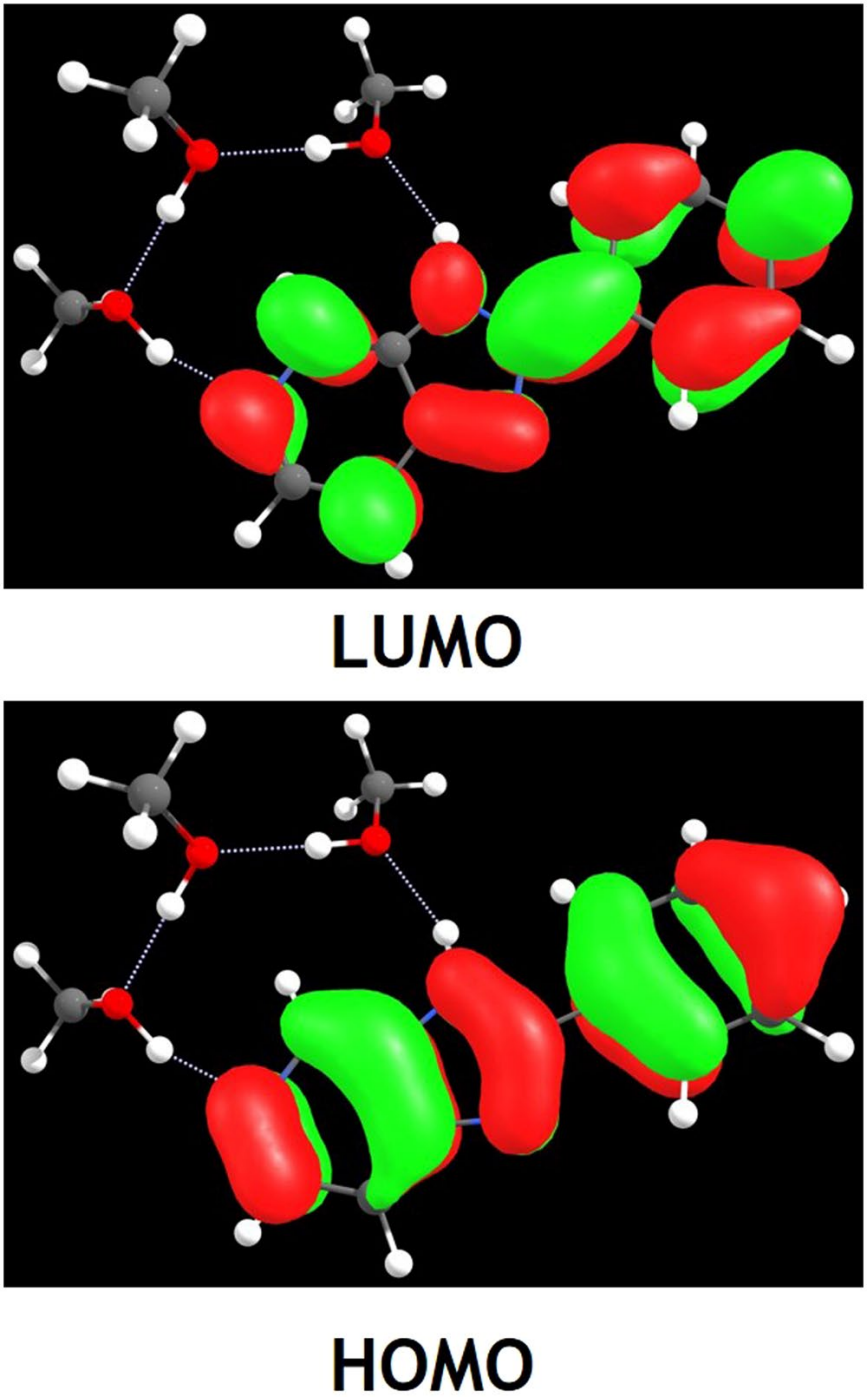
View of frontier molecular orbitals (HOMO and LUMO) for PIP-C-MeOH system.

For further inspecting the effect of the photo-excitation, the excitation energies of the low-lying six excited state about PIP-C-MeOH complex have been calculated. And the absorption and emission spectra of PIP-C-MeOH complex were also calculated using the TDDFT/B3LYP/TZVP theoretical level (see Fig. 3). Results reveal that the absorption peak and fluorescence peak of PIP-C-MeOH are both close to previous experimental results^50^, which demonstrates that the theoretical level used is reasonable for studying PIP-C-MeOH complex. Given the changes about charge distribution upon the excitation, we calculated the frontier molecular orbitals about PIP-C-MeOH and displayed in Fig. 4. The corresponding electronic transition energies, oscillator strengths and compositions have been listed in Table 2. Clearly, the ππ*-type S_1_ state mainly refers to the transition from the highest occupied molecular orbital (HOMO) to the lowest unoccupied molecular orbital (LUMO) with a large oscillator strength 0.7578. It is worth mentioning that all the charge density of MOs are located on PIP-C moiety. Accordingly, it could be confirmed that the hydrogen-bonded wires stay in their ground state in the whole photo-excitation process. The natural bond orbital (NBO) analysis is also done to obtain the quantity of electric charge assigned to the atoms relative to primary atoms along with hydrogen bond wires. Our theoretical results show that the negative charge assigned to N1 is decreased from −0.298 to −0.272 from S_0_ to S_1_ state, meanwhile, the charge assigned to O3 atom is increased from −0.319 to −0.331. Given the N9 atom, it changes from S_0_-state −0.237 to S_1_-state −0.241. It means that the charges are transferred from N1 atom to O3 moiety after the transition from HOMO to LUMO. In addition, one thing could be noted that we have calculated the emission peak of PIP-C-MeOH-PT form is around 365 nm, which is not reported in previous experiment. In effect, this fluorescence 365 nm owns a large oscillator strength about 0.997. Why this emission peak could not be detected will be discussed below.

**Table 2:** Electronic excitation energy (nm), corresponding oscillator strengths and the corresponding compositions for the PIP-C-MeOH complex based on the TDDFT method.

	Transition	*λ*(nm)	*f*	Composition	CI (%)
PIP-C-MeOH	S_0_ → S_1_	288	0.7578	H → L	95.47%
	S_0_ → S_2_	275	0.1261	H-1 → L	90.85%
	S_0_ → S_3_	260	0.0282	H-2 → L	70.38%
				H → L + 1	20.73%

**Figure 5:**
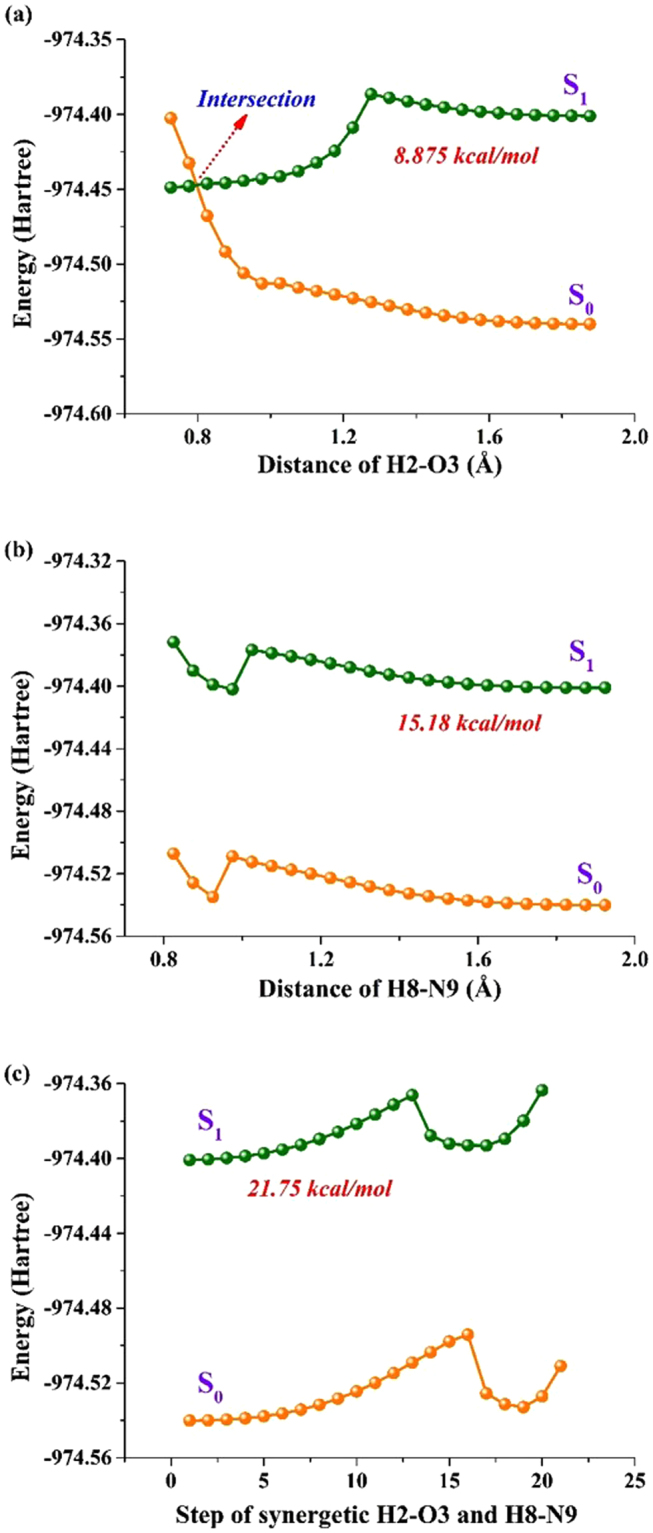
View of three kinds of potential energy curves for PIP-C-MeOH complex in both S_0_ and S_1_ states. (**a**) Closing the distance of H2 and O3 along with N1-H2···O3; (**b**) Closing the distance of H8 and N9 along with O7-H8···N9; (**c**) Synchronous closing H2 and O3 as well as H8 and N9 along with N1-H2···O3 and O7-H8···N9, respectively.

To be best of our knowledge, the theoretical potential energy curves could be a conventional and effective method to investigate the excited state behavior. Thus we theoretically calculated the potential energy curves to further reveal the excited state mechanism for PIP-C-MeOH complex in detail. In this aspect, three kinds of possible conditions of ESIPT have been considered, and the details could be seen in Fig. 5. Obviously, the closing the distance of H2 and O3 along with N1-H2···O3 should be the best way to occur ESIPT process for PIP-C-MeOH. Our calculated potential energy barrier of this manner is just 8.785 kcal/mol, which is not high for finishing the ESIPT reaction. While one thing should be noticed that there is an intersection between S_0_ and S_1_ states. Although TDDFT may be not accurate to locate the conical intersection, it could give a qualitative description between S_0_ and S_1_ states^63–65^. That is to say, even though the ESIPT process could occur, the intersection leads to the nonradiative process from S_1_ to S_0_ state. In other words, we successfully explain why the theoretical 365 nm emission peak could be detected in previous experiment.

## Conclusion

In summary, in this present work, we theoretically investigated the excited state dynamical process for PIP-C-MeOH complex via DFT/TDDFT method. Comparing the bond lengths, bond angles and corresponding IR vibrational spectra, we find that PIP-C-MeOH could transfer the proton H2 firstly, which might open the ESIPT reaction. Further, our work confirms that the PIP-C-MeOH could occur the ESIPT reaction in the S_1_ state, which is different from the attribution of previous experiment. Even though the ESIPT occurs, the emission peak of PIP-C-MeOH-PT form could not be detected due to the intersection between S_0_ and S_1_ states. As a whole, this work puts forward a new nonradiative mechanism for PIP-C-MeOH complex and successfully explain previous experiment.
